# Family carers' experiences and perceived roles in interprofessional collaborative practice in primary care: A constructivist grounded theory study

**DOI:** 10.1111/hex.13828

**Published:** 2023-07-29

**Authors:** Alexandra R. Davidson, Bekhinkosi D. Zigori, Lauren Ball, Mark Morgan, Devanshi Gala, Dianne P. Reidlinger

**Affiliations:** ^1^ Faculty of Health Sciences and Medicine Bond University Gold Coast Australia; ^2^ Centre for Community Health and Wellbeing The University of Queensland Brisbane Australia

**Keywords:** caregiver, carer, chronic disease, multidisciplinary care team, primary health care, qualitative research

## Abstract

**Background:**

Chronic conditions can lead to physical, cognitive and social decline; thus, increasing an individual's dependence on family who assist with activities of daily living. Interprofessional collaborative practice (IPCP), involving two or more health professionals working with the patient and their family, is one model of care for the high‐quality management of individuals with chronic conditions in primary care. Nevertheless, family carers have reported a disconnect between themselves and healthcare providers in previous research. This study aimed to explore the experiences and perspectives of family carers for individuals with chronic conditions, regarding their involvement in IPCP.

**Methods:**

Aspects of constructivist grounded theory methodology were used. Family carers of individuals with chronic conditions were invited to participate in a one‐on‐one, semistructured interview about their experiences with IPCP in the care of their loved one. Interview transcripts were analysed using Charmaz's four‐step iterative process: (1) line‐by‐line coding, (2) focused coding, (3) categorisation of codes and (4) potential theme and subtheme development with memo writing to support each phase of analysis. The research team collaborated on reflexivity exercises, the conceptualisation of categories and the development of themes.

**Results:**

Constructivist data analysis of interviews (average 40 min) with 10 family carers resulted in two themes. (1) Stepping in for my loved one represents the notion that carers take on external roles on behalf of their loved ones (subthemes: working with interprofessional teams, supporting independence and learning as I go). (2) Taking on the carer role, represents the internal factors that influence the external roles described in theme 1 (subthemes: feeling obligated to be involved and changing relationship dynamics).

**Conclusion:**

This study outlines the external actions and internal influences on family carer involvement in an interprofessional team. The required knowledge and support to care for their loved ones is currently learned in an ad hoc manner, and carers' resources should be better promoted by health professionals. Additionally, the relationship dynamics between a carer and their loved one change as the carer becomes more involved in IPCP and influences how and the extent health professionals involve family carers.

**Patient or Public Contribution:**

Carers were the study population involved in this qualitative study. Patient advocates who have chronic conditions, and are informal family carers, were involved in the creation and design of this study, including a review of the research question, participant information sheet and the interview guide.

## 1 INTRODUCTION

Chronic conditions are the leading cause of mortality worldwide,^1^ and are a significant health concern at a global, national and individual level.^2^ In Australia, chronic conditions such as cancer, cardiovascular disease, chronic kidney disease and mental illness are amongst the most common chronic conditions, with almost half (47%) of Australians diagnosed with a chronic condition.^3^ Such conditions are a major contributor towards ill health, disability and death.^2^ The progression of chronic conditions is debilitating and often leads to deterioration in physical, cognitive and social function, often increasing the individual's dependence on carers.^4^, ^5^

Carers are classified as (1) formal carers paid and employed privately or by the health sector, or (2) informal carers defined as family carers, relatives or friends of an individual who may provide care with or without pay.^6^ In Australia, carers are highly prevalent, making up more than 12% of the population.^7^ Carers aid with activities of daily living including personal care, home maintenance, complex medical tasks and transportation, and can spend up to 40 h a week providing care.^8^, ^9^ In Australia, The Carer Recognition Act 2010 states that ‘Carers should be considered as partners with other care providers in the provision of care, acknowledging the unique knowledge and experience of carers’.^10^ However, contrary to this legislative statement, carers have reported a disconnect and poor coordination between themselves and healthcare providers.^5^ In some circumstances, this gap has consequently led to the provision of suboptimal healthcare due to an inability of healthcare providers to consider the individual's holistic needs.^5^

Uncoordinated health care within the community contributes to adverse health outcomes such as reduced quality of life, increased hospital admissions and mortality rates amongst individuals with chronic conditions.^11^ A key model of coordinated care is interprofessional collaborative practice (IPCP), defined as the collaboration and partnership of two or more healthcare providers along with the patient, family, and carers to provide comprehensive health care.^12^ The IPCP model promotes high‐quality, person‐centred care, to meet the quadruple aim of healthcare by improving health outcomes, enhancing the experience of both patient and healthcare provider and reducing healthcare costs.^12^, ^13^, ^14^, ^15^

Several studies have explored patient and healthcare providers' views of the IPCP model.^16^, ^17^, ^18^, ^19^ A recent integrative review that included 48 studies outlined the importance of family and carer involvement in IPCP from the perspective of patients and recommended exploring the family and carer perspective of IPCP in primary care.^20^ Others have investigated the perceptions and experiences of carers for older individuals experiencing ageing‐associated decline or living with intellectual disabilities.^4^, ^21^, ^22^ However, less is known about the views of family carers for individuals living with chronic conditions in the community.^4^ Therefore, this study aims to explore the experiences and perspectives of family carers of individuals with chronic conditions, regarding their involvement in IPCP in Australian primary care.

## 2 METHODS

Qualitative research designs aim to explore the experiences and perspectives of individuals to generate theories that provide an explanation of a particular societal phenomenon.^23^ In this study, we utilised aspects of constructivist grounded theory, consisting of a systematic approach of inquiry to explore a phenomenon of interest with subsequent inductive analysis resulting in the construction of a theory or framework.^24^, ^25^ Grounded in social constructivism and interpretivism,^25^ we explored the experiences and perspectives of carers regarding IPCP and developed a framework that aims to guide primary healthcare professional practice. The researchers also acknowledged that their prior knowledge, experiences and beliefs contributed to the research, through research team reflexive exercises as a method of self‐reflection.^21^, ^26^ During the time of the study, B. D. Z. and D. G. were novice researchers and were trained and guided by researchers experienced in constructivist grounded theory methods A. R. D., D. P. R., M. M. and L. B. A. R. D, D. P. R. and L. B. are accredited practising dietitians, and M. M. is a practicing general practitioner. This study is part of a larger qualitative inquiry in A. R. D's PhD dissertation. Ethical approval was obtained from Bond University Human Research Ethics Committee Ref: AD02910.

We utilised ongoing, heterogenous purposeful sampling,^27^ of 10–15 participants to reach theoretical sufficiency.^28^ Ongoing sample evaluation occurred during the recruitment stages to ensure that there was an equal distribution of family carers that were (1) caring for individuals with a variety of chronic conditions for a diverse representation, and (2) representative of different relationship structures, for example, spouse, parent or child of the individual being cared for.

Potential participants were screened against inclusion criteria before being interviewed: >18 years of age, an informal carer: a family member providing unpaid care for an individual with a chronic condition, and English‐speaking.^29^ Participants were recruited via local primary health networks, professional networks of researchers, a sister study exploring the experiences of patient advocates with chronic conditions,^18^ and snowballing where participants were encouraged to pass on study information to family and friends.^30^ Invitation emails were sent to interested participants who replied directly to the primary researcher either by providing electronic mail consent or rejecting the invitation.

B. D. Z. conducted one‐on‐one interviews using a semistructured guide with open‐ended questions. A. R. D. listened to each of the interviews and met with B. D. Z. to discuss interviews and assist with memo writing. Due to the unforeseeable nature of COVID‐19 and social distancing guidelines, the interviews were conducted via video communication (Zoom).^31^ The interview guide was developed using key literature, IPCP frameworks and research group discussions.^1^, ^22^ A condensed version of the interview guide is outlined in Table 1, and the full version is provided in Supporting Information: File 1. The interview guide was developed in a related study,^18^ amended to suit family carers rather than patients themselves and then piloted with a patient advocate who cared for his mother‐in‐law.^24^, ^25^ Interviews were video and audio recorded and deidentified during verbatim transcription to ensure participant anonymity.^21^ Audio recordings were transcribed by B. D. Z. and checked for accuracy by D. G.

**Table 1:** Semistructured interview question guide.

Number	Type	Key question
1	Introductory questions—ice breaker	Can you start by telling me a bit about yourself?
2	Complex/chronic disease perspectives	Could you tell me a bit about the condition of the person you care for?
3	Case questions Prompt: Can you explain what you mean by ‘x’?	Facilitators to involvement Could you tell me about a specific time or experience you were more involved in the care/treatment of the person you care for?
		Barriers to involvement Could you tell me about a specific time you felt you were NOT involved in the care/treatment of the person you care for?
4	Roles	What do you perceive to be your role in team care?
5	Sampling/recruitment	Do you have any family or friends who are also carers and may be interested in being interviewed?

Recruitment, data collection and data analysis were a nonlinear iterative process. Ending that process was determined by the theoretical sufficiency of the data set, whereby the research team determined they had sufficient data to meet the aims of the study.^24^, ^25^ Data analysis commenced immediately after each interview, and was guided by Charmaz's methods through four key steps: (1) initial line‐by‐line coding, which involved the researcher coding each line of the transcript, (2) focused coding which involved separating, sorting and synthesising initial codes, (3) categorisation of focused codes and (4) theoretical coding to develop potential subthemes and themes.^24^, ^25^ Ultimately, the themes and theories were constructed from the data.^24^, ^25^ Steps 1 and 2 of data analysis were led by B. D. Z. and supported by A. R. D., step 3 was led by B. D. Z. and supported by A. R. D., D. G. and D. P. R. The final and fourth step of development of subthemes and themes was led by B. D. Z. and A. R. D. and supported by the other researchers. Constant reflexive memo writing and regular group reflexive discussions were conducted throughout the data analysis process to improve the rigour and quality of the analytic process.^26^ This manuscript was written using the Standards of Reporting Qualitative Research checklist, provided in Supporting Information: File 2.

## 3 RESULTS

### 3.1 Sample characteristics

**Figure 1:**
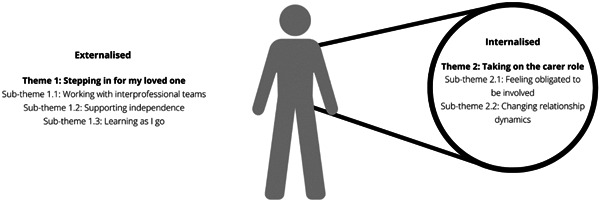
Experiences and perspectives of carers of interprofessional collaborative practice (IPCP) in primary care: This visual framework represents the interconnected extrinsic and intrinsic factors that impact the carer's role in IPCP.

Eleven informal family carers were recruited, and 10 were interviewed. Interviews went for an average of 40 min. One participant initially agreed when recruited via snowballing but later rescinded due to time constraints when sent a formal email invitation. Six female and four male informal family carers of people with a variety of chronic conditions were interviewed (Table 2). Two main themes were developed: (1) stepping in for my loved one and (2) taking on the carer role (Figure 1).

**Table 2:** Demographics for all participants.

Participant number	Carer description	Individuals being cared for and their condition
1	Son‐in‐law	Woman with dementia and co‐morbidities
2	Widowed husband	Woman with chronic kidney disease and co‐morbidities
3	Wife	Man with head and neck cancer
4	Wife	Man with terminal brain cancer
5	Wife	Man with head and neck cancer
6	Father	Young man with spina bifida
7	Wife	Man with oesophageal cancer
8	Daughter	Woman with dementia
9	Son	Man with chronic obstructive pulmonary disease
10	Mother	Man with spinal cord injury

#### 3.1.1 Theme 1: Stepping in for my loved one

This theme was constructed around the core notion that carers took on external roles when interacting with interprofessional teams as a proxy for the individual they were caring for (their loved one), because of reduced capacity. The external family carer roles were captured in three subthemes: working with interprofessional teams, supporting independence and learning as I go.

*Subtheme 1.1: Working with interprofessional teams*: In caring for their loved ones, carers developed ongoing collaborative working relationships with healthcare teams who carers often consulted with on behalf of their loved ones. They perceived they had a coordinator role within the IPCP team, which involved managing correspondence, providing information, negotiating treatment requirements, advocating on their loved one's behalf, collecting prescriptions and booking appointments with healthcare team members.

If something is not right or not working, then you've gotta‐you've gotta look into it and you've got to you've gotta get appointment with somebody, some professional there that can help you. (Participant 5, partner of man with palliative cancer diagnosis)

Carers described that they acted as a conduit for information when communicating with the IPCP team, including the medical and social history of their loved ones. This was seen as a duty, either due to the loved one becoming unreliable in recalling information, or to reduce the burden on them. They were also involved in receiving and implementing the recommendations of the IPCP team and advocating when recommendations may not be suitable for and/or were poorly understood by their loved ones. To ensure this role was fulfilled, they described scribing notes during appointments and sharing these with relevant other healthcare providers.

I would put it [carer's role] down as the coordinator/project manager situation…initiating the emails to the different specialists, chasing them up…getting answers. (Participant 2, Widowed husband of woman with chronic kidney disease & comorbidities)

Carers described feeling included or excluded in IPCP at different stages of their loved one's healthcare journey. Although carers expressed that they felt included in IPCP, there were some instances where they felt excluded either by their loved ones or the healthcare team. Loved ones were perceived by the carer to intentionally not disclose medical information or updates that the carer was not formally included in. Thus, the exclusion came from the loved one and not the IPCP team:

…they've [health professionals] always included me, and they're always happy to…He's [his son] the one keeping me out of any loops that I'm not in, because the health care professionals have all been pretty good. (Participant 6, Father of young man with spina bifida)

Conversely, feelings of exclusion by the IPCP team were described by carers when they felt overlooked in appointments they attended with their loved ones. The focus was on the loved one as the patient, and despite the carer's role as coordinator of care, the IPCP team was perceived by the carer to have overlooked them. Carers themselves at times felt overburdened and needed support from the IPCP team, but did not receive this support:

I know like he's not happy with you know whatever she's recommending, but she never had the courtesy to ask him ‘so what do you want?’ She never had the courtesy to approach me and ask 'so what do you think of this? (Participant 10, Mother of man with spinal cord injury)

…and there were times throughout the journey that the carer is forgotten about, and it's all about the patient. (Participant 3, Wife of man with head and neck cancer).

*Subtheme 1.2: Supporting independence*: As time went on and carers and their loved ones got older, carers needed to adapt to support their loved one's desire to maintain their independence. This threat to their loved one's autonomy may have been due to deterioration in health status as part of the nature of their loved one's chronic conditions, such as a decline in cognitive or physical capabilities, or their child becoming an adult. Carers tried their best to support as much self‐sufficiency of their loved ones as they could. While there was frustration when they wanted their loved ones to accept their care, it was declined, particularly in instances where their loved one's condition worsened, such as cancer or dementia, but there was particularly strong independence displayed:

Um, [my husband] is very independent… he is not always very open to being told, ‘OK, I'll be doing it’. (Participant 4, Wife of man with terminal brain cancer)

For other carers, this independence represented the individual taking on responsibility for their health and they welcomed it:

I'm ready to have [my son] step up I think and be a young adult. That independence would be really good. (Participant 6, Father of young man with spina bifida).

*Subtheme 1.3: Learning as I go*: Carers described the provision of care as a journey that required ongoing self‐education about the medical condition/s and treatment requirements of their loved one. This encompassed learning about the diagnosis and natural progression of the condition. Carers would also upskill, where appropriate, on complex medical tasks such as administering medications, operating medical equipment and navigating pathways for accessing primary healthcare IPCP teams. For example, this man caring for his wife with chronic kidney disease learned about how to best support her dialysis:

I was progressively getting more and more involved to understand what the treatment was and in terms of um, setting up on and doing the stock control of getting the fluids in. (Participant 2, Widowed husband of woman with chronic kidney disease and comorbidities).

Carers were resourceful in seeking reliable sources of information. They described participating in extra training, utilising other family members and the IPCP team to seek out the information they needed to be able to best support their loved one's care needs. One participant indicated that she ‘relies only on medical professionals’ to translate medical jargon into layman's terms and this meant relying on a family member for information.

…it helps that [my husband's] brother is a GP [General Practitioner]. So, when I wanted to ask something, I would ask him. (Participant 4, Wife of man with terminal brain cancer)

Carers also described the need for health professionals to educate them in order for them to be able to care for their loved ones.

…Well, a lot of the time, we're the ones actually…giving the care. You know, there's only a limited interface with the care team. So, we're unqualified caregivers sort of thing. So, we need to be taught how to do things. (Participant 1, Son of woman with dementia)

#### 3.1.2 Theme 2: Taking on the carer role

This theme describes the internalisation of the carer role and the internal factors that participants spoke of as underpinning the external roles when interacting with the IPCP team described in theme 1. These internalised thoughts and feelings are captured in the subthemes: feeling obligated to be involved and changing relationship dynamics.

*Subtheme 2.1: Feeling obligated to be involved*: When carers were asked about their involvement in IPCP, they said it was their duty and described that they felt obliged to take on the carer role. Their responsibilities as a family carers were spoken about as being thrust upon them unexpectedly with little choice or alternatives. Carers noted that health professional team members relied on them for information and communication both at the time of consults and for follow‐up. The carer role was assumed by the family member without consideration of their own needs or preferences, and without clear boundaries. The expectations came from both the loved one, and the healthcare team.

Sometimes I kind of feel like I'm not doing enough, but I don't know what there is. Like what else I could be doing to do more kind of thing. But also at the same time, I kind of don't wanna be doing it if that makes sense. (Participant 9, son of man with COPD)

Carers described their role as being a full‐time coordinator, as caring for their loved one was 24 h a day, 7 days a week. They felt their loved one's care would suffer without them coordinating tasks such as correspondence with the IPCP team, medication management, special diet preparation and adhering to complex management tasks. Additionally, carers assisted with basic activities of daily living such as cooking, shopping and transportation. Despite family, carers began to see their role as an occupation.

I regard my development as a carer and consumer advocate as a post‐business career…people have told me that ‘you're a professional carer’. (Participant 2, Widowed husband of woman with chronic kidney disease & comorbidities)

Carers described accompanying their loved ones during consultations with IPCP team members for support, often due to their loved ones being unable to remember or cope with the information provided. By virtue of being physically present within the consultation room, carers felt they had no option but to be involved in IPCP.

We reached a stage where I had to go in and talk to all the health professionals with her because she couldn't… she used to get too nervous because she couldn't find words. So, she got nervous, and she ended up not being able to speak at all. (Participant 8, Daughter of woman with dementia)

*Subtheme 2.2: Changing relationship dynamics*: Carers described a transition in relationship dynamics with their loved ones. The progression of chronic conditions brought about new roles and responsibilities for the carer thus, changing how the carer and their loved one related to each other. Carers were partners with their loved one, a parent or an adult child or in‐law. The roles and responsibilities of being a coordinator with a team of professionals, advocating for their loved ones within the IPCP team and home care provider where they assisted with activities of daily living and medical tasks were seen as additional to their family role. This impacted their relationship to be more functional than emotional, and the functional responsibilities created tension.

I didn't really get any support as a carer‐ from that team care, you know. It was all about him….there's not really like support for carers like emotionally. (Participant 7, Wife of man with cancer)

For the carers, these new roles and responsibilities seemed to transform romantic partners into patients and parents into children. Where the care originated in childhood, there was conflict as the child sought increasing independence as an adult, yet still required care from their parents. These demanding roles shifted the relationship dynamics as the carer obligation began to overtake the original relationship.

I seriously moved into the carer type role, when my wife, was at a moderately advanced stage of renal kidney failure…progressively, picking up, and getting involved with more and more of her medical conditions and treatment requirements… you could say I became the dialysis nurse in operating the dialysis machine… (Participant 2, Widowed husband of woman with chronic kidney disease & comorbidities)

## 4 DISCUSSION

This study explored the experiences and perspectives of carers for individuals with chronic conditions regarding their involvement in IPCP in primary care. The two main themes (stepping in for my loved one and taking on the carer role) and five subthemes that were constructed suggest that in most circumstances family carers felt involved in IPCP but with some exceptions. There were some instances, such as episodes where treatment plans changed rapidly, or where there was a decline in their loved one's cognition, where carers felt neglected and excluded from team care. Carers suggested that their needs could be better met if the primary healthcare team: (1) was more aware of resources that support carers to contribute to IPCP and signposted these to carers and (2) understood the changing relationship dynamic between the carer and their loved one and how this influences the carer's level of involvement.

The themes and subthemes developed in our study strongly suggest the need for primary health care providers as a team to recognise the needs of carers to support their ongoing involvement in IPCP. Family carers in our study have described an important role being thrust upon them suddenly, a role that has continuously evolved and affected their important relationships. The chronic nature of their loved ones' conditions makes it almost inevitable that the carers' roles will evolve as the condition progresses,^4^ and the number and type of health professionals within the IPCP team will also change over time.^32^ Maintaining the motivation of family carers to be resilient and continue their team role for the longer term should be an additional goal for the primary care team.

Interestingly, other authors have explored the role of self‐determination theory in the motivation of informal carers including a recent integrative review.^33^ Self‐determination theory specifically considers social context to identify factors that support or hinder one's ability to thrive.^34^ The findings of this review confirmed that the theory has utility in promoting and supporting carer motivation and well‐being.^35^ Self‐determination theory describes human motivation through the satisfaction of three basic psychological needs: autonomy, relatedness and competence,^34^ each of which are evident through the themes of our study. Our findings will now be explored in relation to self‐determination theory.

The concept of autonomy, whereby one feels able to make choices for oneself, was evident in our study throughout the two themes and the subthemes. Self‐determination theory identifies that motivation sits on a continuum of varying autonomy, ranging from autonomous (associated with the individual undertaking valued actions and deriving satisfaction from them) to control by external forces (whereby actions are undertaken to receive a reward or avoid some kind of punishment including feelings of guilt).^36^ In our study, it was clear that carers experienced pressure through healthcare teams consulting directly with them to respond on behalf of their loved ones, as well as through their sense of duty to their loved ones. Carers in our study described a sense of duty and obligation that was overwhelming and felt their involvement in team care was often thrust upon them, reflecting external pressures, and a finding consistent with previous research.^37^ Decreasing the independence of their loved one also invoked this sense of obligation which contributed to the carer's sense of burden. Further, carers were charged with supporting the autonomy of their loved one potentially at the expense of reducing their own autonomy to provide care in the way they preferred. Therefore, primary healthcare IPCP teams can support carer autonomy by explicitly identifying the extent to which family members are willing to be a family carer, and what this may look like in terms of roles and responsibilities of the family carer.

Relatedness, or a feeling of social connection, is described as supporting autonomy and one's intrinsic motivation to perform a role within self‐determination theory.^34^ Relatedness was clearly important to the carers in our study, who were affected by the changing relationship with their loved one as they increasingly assumed the role or occupation of ‘carer’. There were obvious tensions in the carer‐loved relationship as a result of the family carer arrangement. Further, the observation that at times they felt forgotten by the healthcare team points to a need for the carer to feel a connection within the IPCP team in addition to maintaining their relationship with their loved one. Our finding that carers were already feeling included in IPCP and feeling part of a team of people caring for their loved ones, is encouraging and promotes relatedness. Such inclusion aligns with the IPCP model, which is based on team care between the healthcare provider, the patient and their family and carer(s).^12^ This contrasts with earlier studies that reported a disconnect between healthcare providers and carers resulting in fragmented care.^38^ It has been suggested that health professionals actively showing respect and empathy towards carers is one way to meet carer‐relatedness needs.^39^

Competence, that is, the need to master a role and feel effective, is the third tenet of motivation described within self‐determination theory,^34^ which was evident in our findings. The subtheme ‘Learning as I go’ particularly captured the need for competence amongst our family carer participants. They described how they identified the need for self‐education about the condition and its progression, as well as learning clinical skills and healthcare communication. Previous literature also highlights that, due to a gradual capacity decline, individuals living with a chronic condition may become unable to effectively provide and receive healthcare information, thus, unable to be completely independent in managing their own care.^8^ The critical role played by informal carers in IPCP as key links in communication between the patient and the healthcare provider have been recognised.^40^ In a large meta‐analysis exploring health professional support for *patient* autonomy, the perception of competence explained the largest proportion of variance in health outcomes when compared with autonomy and relatedness concepts.^35^ This suggests that efforts to support carer competence may be an effective way for the primary health team to improve outcomes for both the patient and their family carer.

The subtheme of ‘Learning as I go’ reflected carers' resourcefulness in seeking reliable information from health professionals regarding their loved one's treatment. This theme also illustrates carers' commitment to ongoing self‐education of their loved one's diagnoses and needs. These findings demonstrate that both healthcare professionals and carers must establish a working relationship whereby the carer is provided with resources and education by health professionals, and the carer actively seeks information.

Our study has provided insights into the perspectives of family carers that have implications for primary care health professionals involved in IPCP. The three tenets of self‐determination theory provide a framework for action areas for the primary care team to support carers' involvement in IPCP—autonomy, relatedness and competence. Autonomy can be promoted by providing carers and their loved ones with options for care outside of the family so that the carer has a genuine choice about the roles they adopt. Involving carers and their loved ones in decisions about treatment options can also strengthen carer autonomy. Relatedness can be promoted through the team actively including the carer as part of IPCP and acknowledging their important role. Consideration of carer's burden, and the impact of caring on their relationship with their loved one, is also important. It is widely acknowledged that carer burden, defined as an overall emotional, physical, psychological, behavioural and financial toll, is highly prevalent amongst family carers.^41^ Healthcare providers should acknowledge not only their patient's medical conditions, but the potential burden on, and burnout of, the carer. The primary care team could consult with the carer, without their loved one present, and screen for carer's burden to ensure adequate psychological support and appropriate referrals for social support as needed.^42^ By reducing the carer burden, carers are supported to continue to be effective in team care.^41^ Finally, competence is promoted through directly educating carers, providing recommendations for training or reputable information, and acknowledging the carer's knowledge and skills with respect.^39^ Recent evidence further supports that it is the healthcare provider's role to educate carers regarding the complex health status of their loved ones, including the provision of resources and information regarding their diagnosis, prognosis, and care needs.^43^

### 4.1 Strengths and limitations

A strength of this study is the representation of family carer views and the perception of their role in IPCP, a gap identified in previous literature.^20^ Heterogenous sampling was used to achieve diversity of the relationship structures of carers to their loved ones, and chronic conditions of loved ones. However, there were more relationship structures of people caring for spouses and their elderly parents, compared to parents caring for their adult child with a chronic condition or disability. Additionally, the results are only a representation of the experiences and perspectives of informal carers and not formal carers. Formal carers, including support workers, are also essential in the care and management of people with chronic conditions and disabilities who have higher care needs that may not be able to be fulfilled by family carers. Future research exploring the experiences and perspectives of formal carers regarding their involvement in IPCP would assist in understanding the support needs of formal carers for their involvement in IPCP teams in the primary care setting. The sample size was limited to ten participants due to time constraints and the need for rich data to fulfil the rigour required for constructivist grounded theory analysis. Others have suggested a minimum sample size of 15 participants as common for grounded theory research designs.^28^ However, after the eighth interview was conducted and analysed, we determined we had enough data for theoretical sufficiency. A potential limitation was that data collection took place during the COVID‐19 public health restrictions. We included participants who may have been carers in the past as well as those who were current carers. Our interest was in gaining perspectives from carers on what they saw as their role in IPCP, as well as their general experience of IPCP as a carer, rather than leading their responses with explicit COVID‐19 questions.

## 5 CONCLUSION

Carers in this study perceived that collaborative practice often occurs in primary care between the healthcare provider, the carer and their loved one. However, there are instances where carers feel excluded in team care suggesting that collaborative practice could be further strengthened to ensure the highest quality of care for the loved one. Carers described being proactive in seeking out health information and education to better support their loved ones. However, not all carers may have this capacity, thus primary healthcare providers should be fulfilling the role of educators and providing resources to better support carers. Regular screening for carer burden and providing support where necessary should also be led by primary healthcare professionals. Reducing the carer burden sustains the carer's role in IPCP thus protecting the loved one from adverse effects. This study has highlighted that the role of family carers in IPCP in the primary care setting includes hidden roles such as advocacy for their loved one and performing a ‘job’ as the full‐time coordinators of care. Health professionals should support carers by maximising their involvement in decision‐making, providing empathy and respect for the carer's role and providing education to carers to perform their role. Additionally, health professionals should acknowledge the changing relationships between a carer and their loved one and offer direct care to the carer to identify and manage the mental health impacts of their role.

## AUTHOR CONTRIBUTIONS

Alexandra R. Davidson, Lauren Ball, Mark Morgan and Dianne P. Reidlinger contributed to the study conceptualisation and design. Bekhinkosi D. Zigori conducted data collection. Bekhinkosi D. Zigori and Alexandra R. Davidson led data analysis with assistance from all other authors. Bekhinkosi D. Zigori wrote the first draft of the manuscript, and Alexandra R. Davidson and Dianne P. Reidlinger wrote the revised version of the manuscript. All authors read and approved the manuscript before submission.

## CONFLICT OF INTEREST STATEMENT

Bekhinkosi D. Zigori was employed at Gem Complete Health Services, from which one participant was recruited. The remaining authors declare no conflict of interest.

## ETHICS STATEMENT

Ethics approval was gained from the Bond University Human Research Ethics Committee, ethics approval number AD02910. Family carers provided written informed consent before the commencement of interviews.

## Supporting information

Supporting information.

Supporting information.

## Data Availability

Research data are not shared due to ethical/privacy reasons. The data are not publicly available due to privacy or ethical restrictions.
