# Supplementary material for: Family carers' experiences and perceived roles in interprofessional collaborative practice in primary care: A constructivist grounded theory study
Source: Health Expect. 2023 Jul 29;26(6):2302–11. doi: 10.1111/hex.13828 (PMC10632645; doi:10.1111/hex.13828)
Supplement: Supplementary file 2 — Supporting information. [file HEX-26--s001.docx]

Article Title: Family carers’ experiences and perceived roles in interprofessional collaborative practice in primary care: A constructivist grounded theory study

Supplementary File 2: Standards for Reporting Qualitative Research (SRQR) Checklist

Reference: O'brien, B. C., Harris, I. B., Beckman, T. J., Reed, D. A. & Cook, D. A. 2014. Standards for reporting qualitative research: a synthesis of recommendations. Acad Med, 89, 1245-51.

| No. | Topic | Item | Manuscript Page and Line Number |
| --- | --- | --- | --- |
| **Title and Abstract** | | | |
| S1 | Title | Concise description of the nature of and topic of the study identifying the study as qualitative or indicating the approach (e.g., ethnography, grounded theory) or data collection methods (e.g., interview, focus group) is recommended | Page 1, line 1 - 2 |
| S2 | Abstract | Summary of elements of the study using the abstract format of the intended publication; typically includes background, purpose, methods, results, and conclusions | Page 1, line 3 – 30 |
| **Introduction** | | | |
| S3 | Problem formulation | Description and significance of the problem/phenomenon studied; review of relevant theory and empirical work; problem statement | Page 3, line 47 - 73 |
| S4 | Purpose or research question | Purpose of the study and specific objectives or questions | Page 3, line 74 – Page 4, line 82 |
| **Methods** | | | |
| S5 | Qualitative appoach and research paradigm | Qualitative approach (e.g., ethnography, grounded theory case study, phenomenology, narrative research) and guiding theory if appropriate; identifying the research paradigm (e.g., postpositivist, constructivist/interpretivist) is also recommended; rationale | Page 4, line 84 - 90 |
| S6 | Researcher characteristics and reflexivity | Researchers’ characteristics that may influence the research, including personal attributes, qualifications/experience, relationship with participants, assumptions, and/or presupposition; potential or actual interaction between researchers’ characteristics and the research questions, approach, methods, results and/or transferability | Page 4, line 90 - 96 |
| S7 | Context | Setting/site and salient contextual factors; rationale | Page 4, line 89-90 |
| S8 | Sampling strategy | How and why research participants, documents, or events were selected; criteria for deciding when no further sampling was necessary (e.g., sampling saturation); rationale | Page 4, line 99-103 |
| S9 | Ethical issues pertaining to human subjects | Documentation of approval by an appropriate ethics review board and participant consent, or explanation for lack thereof; other confidentiality and data security issues | Page 4, line 96-98, and Page 5, line 120-121 |
| S10 | Data collection methods | Types of data collected; details of data collection procedures including (as appropriate) start and stop dates of data collection and analysis, iterature process, triangulation of sources/methods, and modification of procedures in response to evolving study findings; rationale | Page 5, line 112-122 |
| S11 | Data collection instruments and technologies | Description of instruments (e.g., interview guides, questionnaires) and devices (e.g. audio recorders) used for data collection; if/how the instrument(s) changed over the course of the study. | Page 5, line 115-121 Table 1 and Supplementary File 1 |
| S12 | Units of study | Number and relevant characteristics of participants, documents, or events included in the study; level of participation (could be reported in results) | In Methods, Page 4, line 99-103, and in Results, Page 6, line 140-143 and Table 2 |
| S13 | Data processing | Methods for processing data prior to and during analysis, including transcription, data entry, data management and security, verifcation of data integrity, data coding, and anonymization/deifendification of excerpts | Page 5-6, line 124-137 |
| S14 | Data analysis | Process by which inferences, themes, etc., were identified and developed, including the researchers invovled in data analysis; usually references a specific paradigm or approach, rationale | Page 4, line 85-90  Page 5-6, line 126-136 |
| S15 | Techniques to enhance trustworthiness | Techniques to enhance trustworthiness and credibility of data analysis (e.g., member checking, audit trail, triangulation); rationale | Page 4, line 90-92, and Page 5, line 113-114, and 121-122. |
| **Results/findings** | | | |
| S16 | Synthesis and interpretation | Main findings (e.g., interpretations, inferences, and themes); might include development of a theory or model, or integration with prior research or theory | Page 6, line 138 – Page 11, line 285 |
| S17 | Links to empirical data | Evidence (e.g., quotes, field notes, text excerpts, photographs) to substantiate analytic findings | Quotes provided for each theme and sub-theme |
| **Discussion** | | | |
| S18 | Integration with prior work, implications, transferability, and contribution(s) to the field | Short summary of main findings; explanation of how finding and conclusions connect to, support, elaborate on, or challenge conclusions of earlier scholarship; discussion of scope of application/generalizability; identification of unique contribution(s) to scholarship in a discipline or field | Page 11, line 286 – Page 14, line 384 |
| S19 | Limitations | Trustworthiness and limitations of findings | Page 15, line 385-406 |
| **Other** | | | |
| S20 | Conflicts of interest | Potential sources of influence or perceived influence on study conduct and conclusions; how these were managed | Title page |
| S21 | Funding | Sources of funding and other support; role funders in data collection, interpretation, and reporting | Title page |
